# Commentary: Monocyte and macrophage lipid accumulation results in down-regulated type-I interferon responses

**DOI:** 10.3389/fcvm.2022.1086136

**Published:** 2023-01-09

**Authors:** Fang Li, Hanrui Zhang

**Affiliations:** Cardiometabolic Genomics Program, Division of Cardiology, Department of Medicine, Columbia University Irving Medical Center, New York, NY, United States

**Keywords:** atherosclerosis, lipids, macrophages, inflammation, immunometabolism, foam cells

Macrophages possess remarkable phenotypic and functional plasticity in response to micro-environmental stimuli (1). In atherosclerosis, macrophages sequester extracellular modified lipids, which accumulate as cytoplasmic lipid droplets, to form foamy macrophages that are crucial during all stages of the disease (2). In particular, the death of foamy macrophages contributes to the development of the lipid core and unstable plaques associated with an increased propensity to plaque rupture and acute cardiovascular events (3).

It has been thought that foamy macrophages drives chronic inflammatory responses, yet recent literature demonstrates that foamy macrophages are in fact less inflammatory than their non-foamy counterparts in the plaque (4). Mechanistically, cholesterol loading of macrophages *in vitro* results in the activation of liver X receptor (LXR), which upregulates genes involved in reverse cholesterol transport and exerts anti-inflammatory effects (5). Myeloid LXR deficiency induces inflammatory gene expression in foamy macrophages and accelerates atherosclerosis (6). LXRs are activated by oxysterols formed in response to increased intracellular cholesterol levels. The foamy peritoneal macrophages of atherosclerosis-prone *Ldlr*^−/−^ mice fed with a high-cholesterol/high-fat diet also accumulate desmosterol, an LXR ligand with both LXR-dependent and independent effects (7). Desmosterol activates LXR target genes, inhibits sterol regulatory-element binding proteins (SREBPs) target genes, alters fatty acid metabolism, and suppresses genes involved in inflammatory responses, e.g., *Il1b, Cxcl9*, and *Cxcl10* (7). Furthermore, peritoneal macrophages from *Ldlr*^−/−^ mice fed with a high-fat diet had lower pentose phosphate pathway (PPP) metabolites than mice fed with a normal diet upon lipopolysaccharide stimulation, contributing to diminished lipopolysaccharide-induced production of pro-inflammatory genes (8). Activation of LXR does not affect PPP metabolites, supporting LXR-independent mechanisms of diminished inflammatory phenotypes of foamy macrophage (8). Despite these important progresses, there remains a major knowledge gap in the mechanistic links between lipid accumulation and immune response modulation.

In this issue of *Frontiers in Cardiovascular Medicine*, leveraging transcriptomic data analyses, Willemsen et al. moved one important step forward toward addressing how foamy cells are less inflammatory (9). The study analyzed four public and newly generated transcriptomic datasets, including: (1) bone marrow-derived macrophages (BMDMs) with or without loading of acetylated low-density lipoprotein (ac-LDL); (2) peritoneal macrophages derived from wildtype and *Apoe*^−/−^ mice; (3) human monocytes from familial hypercholesterolemia patients and healthy controls; 4) BMDMs with or without the treatment of LXR-agonist GW3965. The results support that ac-LDL loading in murine and human macrophages specifically suppressed interferon-β (IFN-β) secretion and the expression of IFN-stimulated genes (ISGs), but not many other pro-inflammatory genes (9). The downregulation of ISGs could be rescued by exogenous IFN-β supplementation. LXR activation suppressed the expression of ISGs, resembling the effects of lipid loading (9). Monocytes of familial hypercholesterolemia patients also show a deactivated IFN signature which was restored by lipid-lowering therapy (9). The results further strengthened the notion that lipid-loaded foamy macrophages of mice and humans are less inflammatory and the specific involvement of perturbed type-I IFN responses (9).

Many questions remain unanswered. Beyond the role of LXR activation (9), if and how additional molecular mechanisms may be involved in suppressing the expression of ISGs in foamy macrophages remain undetermined. The current analyses highlighted a transcriptomic signature of suppressed IFN-β responses that is consistent in foamy macrophages *in vitro* and *ex vivo*, yet the differences among datasets may provide additional insights. Meta-analysis of RNA-seq data from multiple studies will further reveal the effects of lipoproteins with different modifications in macrophages from distinct origins and organ locations. Integrating transcriptomic data with phenotypic and biochemical characterization will inform the precise identity of foamy macrophage and the extent of lipid accumulation required for the suppression of inflammation. Further, it is unknown why the non-foamy macrophages remain not lipid-loaded in the atherosclerotic plaque; for example, if the foamy macrophages arise as a consequence of a relative increase in phagocytic activity, or if non-foamy and foamy macrophages have a distinct spatial distribution with different degrees of lipoprotein retention (10). The study (9) also provides additional mechanistic and therapeutic implications. First, it will be intriguing to determine if the molecular mechanisms of suppressed inflammation by lipid loading may be leveraged for therapeutic applications. Second, unloading excessive cholesterol to reverse the formation of foam cells represents important therapeutic strategies to decrease atherosclerotic plaque burden. The analysis supports that lipid-lowering therapy reverses IFN-β suppression (9), providing a rationale for testing the combination of lipid-lowering and anti-inflammatory therapies for atherosclerosis regression. Third, while current methods capture a snapshot of foamy macrophage profile, gaining insights into the spatial and temporal signature of foam cell development may further unleash their prognostic value.

More broadly, in addition to atherosclerosis, foamy macrophages are frequently observed in different pathological states, including infectious diseases, multiple sclerosis, and cancer (11–14). Because of the important roles of type I IFNs in host defense against viruses (15), targeting lipid metabolism in monocytes using lipid-lowering treatment to promote anti-viral defense is potentially promising (9). In tuberculosis granuloma, foamy macrophages act as key participants in both sustaining persistent bacteria and contributing to tissue pathology (11). In multiple sclerosis lesions, myelin ingestion by myeloid cells induces a foamy appearance and confers anti-inflammatory function (12). Accumulation of lipids in tumor-associated macrophages (TAMs) elicits an immunosuppressive phenotype (13). Consequently, disrupting lipid droplet formation in TAMs impeded tumor growth in mice (13). Consistently, large foamy TAMs were more frequently found in colorectal liver metastasis patients with worse prognoses than patients with good prognoses (14). Lastly, other cell types, such as dendritic cells and vascular smooth muscle cells, also take up lipids and form foam cells (16–18). The disease- and cell type-specific mechanisms of foam cell biogenesis and function warrant extensive research and may have broad impact.

In summary, this study by Willemsen et al. (9) further motivates the research community to fully dissect the biology of foam cells and their relationship to diseases and to explore the potential role of foam cells as prognostic and therapeutic targets at an immunometabolism level.

## Author contributions

All authors listed have made a substantial, direct, and intellectual contribution to the work and approved it for publication.
